# 2022 Acknowledgment of *Microbiology Spectrum Ad Hoc* Reviewers

**DOI:** 10.1128/spectrum.04724-22

**Published:** 2022-12-21

**Authors:** Christina A. Cuomo

**Affiliations:** a Broad Institute of MIT and Harvard, Cambridge, Massachusetts, USA

## EDITORIAL

The editors of *Microbiology Spectrum* sincerely thank all the reviewers who contributed to the peer review process in 2022. We are grateful for the contributions of over 4,000 reviewers who have contributed their time, effort, and expertise to the journal. We would like to specifically recognize the following *ad hoc* reviewers who have reviewed three or more articles submitted to *Microbiology Spectrum*. Your dedication to the journal and its success is commendable and greatly appreciated.

Aqleem Abbas
Ghulam Abbas
Hamad Abdelhadi
Elsayed Abdelwhab
Nusaibah Abdul Rahim
Asra'A Abdul-Jalil
Tintu Abraham
Leyda Abrego
Chanelle Acheamfour
Maikel Acosta-Zaldívar
Beth Adamowicz
Innocent Afeke
Surya Aggarwal
Christian Ahearn
Din Ahmad Ud
Iqbal Ahmad
Tariq Ahmad
Ayaz Ahmed
Samuel Aitken
Muhammad Akhtar
Marcelo Alarcon
Randy Albrecht
Ana Alcalá
Gajender Aleti
Inas Alhudiri
Hassan Al-Tameemi
Fairoz Al-Wrafy
Amnon Amir
Xinwei An
Thomas Andersen
Neil Anderson
Simon Andrews
Olga Andrievskaia
Andrey Anisimov
Nana Ankrah
Arun Annamalai
Vijay Antharam
Alberto Antonelli
Agustin Aranda
Olatunji Aremu
Francisco Arenas-Huertero
Mehreen Arshad
Michael Asamoah-Boaheng
Asaad Ataa
Joseph Ayariga
Modupe Ayilara
Hamid Badali
Sakcham Bairoliya
Regina Baldini
David Baltrus
Niaz Banaei
Jeffrey Banas
Sulagna Banerjee
Fernando Baquero
Godfrey Barabona
Daniel Barkan
Rodolphe Barrangou
Gili Ben-Nissan
Susan Benson
Ivan Berg
Julien Bergeron
Teresa Bergholz
Luca Bertzbach
Kanchan Bhardwaj
Souvik Bhattacharyya
Bijit Bhowmik
Scott Biering
Pablo Bifani
Gursonika Binepal
Jordan Bisanz
Jorge Blanco
Patricia Bloom
Zachary Blount
Cornelia Blume
Raquel Bonelli
Kenneth Bongulto
Batbileg Bor
Antoni Bordoy
Angela Boysen
Patricia Bradford
Marc Bramkamp
Stephane Bretagne
Barbara Byrne
C. Joaquin Caceres
Feng Cai
Yun Cai
Meredith Carpenter
Marco Cassone
Vincent Cattoir
Ruben Cebrian Castillo
Kaan Çeylan
Jianmin Chai
Martin Chan
Yung-Fu Chang
Som Chatterjee
Santanu Chattopadhyay
Emmanouella Chatzidaki
Jayeshbhai Chaudhari
Eira Chaudhary
Hongbin Chen
Liang Chen
Lianmin Chen
Nanhua Chen
Peigen Chen
Shoudeng Chen
Tingtao Chen
Ying-Tsong Chen
Loo Wee Chia
Kihyuck Choi
Abdul Choudhry
Franklin Wang-ngai Chow
Clifford Clark
Sean Conlan
Nicolás Cordeiro
Valerie Cortez
Lindsey Crawford
Cristian Crisan
Matthew Crotty
Luis Cruz-Vera
Jie Cui
Sophie Darch
Lizbeth Davila-Santiago
Boudewijn De Jonge
Delphine Dean
Justine Debelius
Erick Denamur
Xianding Deng
Berthony Deslouches
Justen Despain
Sanjay Dey
Sourabh Dhingra
Vincenzo Di Pilato
Mederic Diard
Dzung Diep
Francisco Diez-Gonzalez
Ulrich Dobrindt
Yohei Doi
Robert Dorosky
Zhicheng Dou
Charles Dozois
Milena Dropa
Xiang-Dang Du
Yitao Duan
Zoe Dyson
Allison Eberly
Raghda Eldesouki
Melissa Ellermann
Terri Ellis
Melinda Engevik
Javier Escobar-Perez
Shannon Esher Righi
Eduardo Espeso
Dimitrios Evangelopoulos
David Everly
Annette Fagerlund
Rima Fanaei Pirlar
Mazigh Fares
Matthew Faron
Aléia Faustina Campos
Na Fei
Jie Feng
Youjun Feng
Paulo Fernandes
Paul Fey
Laura Filkins
Renee Fleeman
Niels Frimodt-Møller
Jonathan Frye
Songzhe Fu
To Sing Fung
Raj Gaji
László Galgóczy
Emily Gallichotte
Michael Ganzle
Kan Gao
David Gaston
Rahul Gauttam
Savvas Genitsaris
Heather Glasgow
Mark Gonzalez
Lisa Gorski
Friedrich Götz
Steven Gregory
Thomas Grönthal
Mengyao Guo
James Gurney
Kiran Gurung
Jens Hammerl
Mei-Ling Han
Klaus Hantke
Justin Hardick
Christopher Harmer
Fang He
Emma Hernandez Sanabria
Matthew Hernandez
Janet Hindler
Elizabeth Hirsch
Andrew Hogan
Benjamin Horwitz
Amy Howell
Gongzheng Hu
Ching-Ying Huang
Haijiang Huang
Jinhu Huang
Shi Huang
Jan Hubert
Lauren Hudson
Jonathan Hulse
Mohamed Husen Munshi
Francesco Imperi
Ikbal Agah Ince
Hon Ip
Kenichi Ishii
Mary Jackson
Robert Jackson
Mohamed Jamal
Francis Jenney
Che Ok Jeon
Chao Jiang
Guangde Jiang
Tao Jin
Michael Jobling
Peter Jorth
Yuan Jun
Barbara Kahl
Richard Yi-Tsun Kao
Priyanka Kapoor
Krishanpal Karmodiya
Shahrbanoo Keshavarz Azizi Raftar
Yasutoshi Kido
Hirokazu Kimura
Benjamin Koestler
Car Reen Kok
Laurent Kremer
Benjamin Lafrentz
Li-Yin Lai
Ling Ning Lam
Shuai Le
Jintae Lee
Kenichi Lee
Bin Li
Hao-Sen Li
Ningqiu Li
Peng Li
Ruichao Li
Yue-Zhong Li
Qiming Liang
Yi-Chu Liao
Zhuoren Ling
Martin Linster
Jessica Little
Bo Liu
Chengwei Liu
Jianzhu Liu
Qingyun Liu
Tong-Bao Liu
Yong-Xin Liu
Małgorzata Łobocka
Marta Lourenço
Yahai Lu
Junbo Luan
Jose Luis Martinez
Jonathan Lynch
Anup Majumder
Junki Maruyama
Yasufumi Matsumura
Jodi Mcgill
John Mcmullen
Saurabh Mishra
Surabhi Mishra
Brian Mochon
Karina Montero
Tatum Mortimer
James Moy
Shigeki Nakamura
Gopal Naik Nenavath
Anthony Nicola
Beifang Niu
Steven Norris
Dennis Nurjadi
Dele Ogunremi
Mehmet Orman
Lucia Ortiz
Kenji Ota
George O'Toole
Sophonie Oyeniran
Volkan Özenci
Robert Palmer
John Panepinto
Romina Papa-Ezdra
Costas Papagiannitsis
Colin Parrish
Yousong Peng
Minh-Duy Phan
Tung Phan
Marcelo Pillonetto
Daniel Pletzer
Laurent Poirel
Mimi Precit
Henry Puerta-Guardo
Hua-Ji Qiu
Alaa Rabee
Marcela Radice
Rama Ramani
Xiancai Rao
Jeremy Ratcliff
Sylvie Rebuffat
Carla Rodrigues
Eduardo Rodríguez-Román
Denis Roy
Eugene V. Ryabov
Silvia Salatino
Orjan Samuelsen
Kesavardhana Sannula
Maja Šantak
Timothy Satterlee
Audrey Schuetz
Stefan Schwarz
Patrick Secor
Brittany Seibert
Elif Şeker
Robert Shafer
William Shafer
Kiran Sharma
Ekaterina Shelest
Bang Shen
Cong Shen
Anukul Shenoy
Thomas Shinnick
Anton Sholukh
Longlong Si
Lynn Silver
Ramandeep Singh
Kristen Smith
Emily Snavely
Eike Steinmann
George Stewart
Xingmin Sun
Troy Sutton
Gene Tan
Takeshi Tanaka
Asmaa Tazi
Ma Teng
Guo-Bao Tian
Tristan Timbrook
Laura Tipton
Clement Tsui
Christine Turenne
Can Ünal
Daniel Unterweger
Françoise Van Bambeke
Maria Vehreschild
Henrietta Venter
Romain Volmer
Chengming Wang
Hong-Ning Wang
Jun Wang
Junjun Wang
Linqi Wang
Mengcen Wang
Yang Wang
Yongbao Wang
Xiaolin Wei
Zezhang Wen
Stacy White
Katrine Whiteson
Marisa Winkler
Benjamin Wolfe
Gloria Wong
Kuanhui Xiang
Jingfa Xiao
Yonghong Xiao
Jianping Xie
Yanwen Xiong
Tong Xu
Zhenjiang Xu
Chaoyang Xue
Yanlin Xue
Shangxin Yang
Zi-Wen Yang
Peiqi Yin
Sheng-Hua Ying
Dongwan Yoo
Yunsong Yu
Min Yue
Irina Zakharova
Ashraf Zarkan
Martha Zepeda Rivera
Chiyu Zhang
Genwei Zhang
Wen Zhang
Yawei Zhang
Huajun Zheng
Yong Zheng
Jin Zhong
Bin Zhou
Jianli Zhou
Lifeng Zhu
Franz Zingl
Isabelle Zink
Sadri Znaidi

